# Agenda for COVID-19 and long COVID research priorities in Brazil: results of wide consultation and Delphi consensus, 2022-2023

**DOI:** 10.1590/S2237-96222025v34e20240623.en

**Published:** 2025-06-20

**Authors:** Natália Silva Alves, Everton Nunes da Silva, Gabriela Bardelini Tavares Melo, Micaela Alexandra Silva Paulino, Antonia Angulo-Tuesta

**Affiliations:** 1 Universidade de Brasília, Faculdade de Ciências e Tecnologias em Saúde, Programa de Pós-Graduação em Ciências e Tecnologias em Saúde, Brasília, DF, Brazil; 2 Ministério da Saúde, Secretaria de Ciência, Tecnologia e Inovação do Complexo Econômico Industrial da Saúde, Departamento de Ciência e Tecnologia, Brasília, DF, Brazil; 3 Universidade de Brasília, Faculdade de Ciências da Saúde, Programa de Pós-Graduação em Saúde Coletiva, Brasília, DF, Brazil

**Keywords:** Health Research Agenda, Post-Acute COVID-19 Syndrome, COVID-19, Brazil, Exploratory Qualitative Study, Agenda de Investigación en Salud, Síndrome Post Agudo de COVID-19, COVID-19, Brasil, Estudio Cualitativo Exploratorio

## Abstract

**Objective:**

To propose an agenda of COVID-19 and long COVID research priorities, in order to guide government and research funding agencies to optimize health science, technology and innovation resources in Brazil.

**Methods:**

This is a qualitative study, carried out in two stages, between April 2022 and March 2023. In the first stage, a broad consultation was carried out to identify research priorities according to the axes of the COVID-19 Evidence Network to support Decision-making initiative, with 71 participants including researchers, health service managers, health science and technology managers, health professionals and health service users. In the second stage, a consensus was reached on the priorities proposed in the previous stage, using the Delphi method, with a panel of 20 experts on COVID-19 in the first round and 18 in the second round.

**Results:**

In the broad consultation, 186 priority lines of research on COVID-19 were received and consolidated into 161 research lines. Of these, 139 achieved consensus in the first round of the Delphi method, and a further 40 lines were received and included in the second round for consensus. The proposed agenda has 179 research lines on COVID-19. The predominant themes were evaluation, COVID-19 impact and sequelae, long COVID-19, mental illnesses and immunosuppression. The child population was of greatest interest.

**Conclusions:**

This study demonstrated high levels of agreement among participants on COVID-19 and long COVID research priorities in Brazil.

## Resumen

**Objetivo:**

Proponer una agenda de prioridades de investigación sobre Covid-19 y post-Covid, con el fin de orientar a los gobiernos y a los organismos de financiación de la investigación para optimizar los recursos en ciencia, tecnología e innovación en salud en Brasil.

**Métodos:**

Se trata de un estudio cualitativo, realizado en dos etapas, entre abril de 2022 y marzo de 2023. En la primera etapa se realizó una amplia consulta para identificar prioridades de investigación según los ejes de la *COVID-19 Evidence Network to support Decision-making initiative*, con 71 participantes entre investigadores, gestores de salud y de ciencia y tecnología, profesionales y usuarios de servicios de salud. En la segunda etapa se consensuaron las prioridades propuestas en la etapa anterior, utilizando el método Delphi, con un panel de 20 expertos en Covid-19 en la primera ronda y 18 en la segunda ronda.

**Resultados:**

En la consulta amplia se recibieron 186 líneas prioritarias de investigación sobre Covid-19 que se consolidaron en 161 líneas. De ellas, 139 alcanzaron consenso en la primera ronda del método Delphi, y otras 40 líneas fueron recibidas e incluidas en la segunda ronda para consenso. La agenda propuesta cuenta con 179 líneas de investigación sobre Covid-19. Predominaron los temas de valoración, impacto y secuelas del Covid-19, Covid-19 prolongado, enfermedades mentales e inmunosupresión. La población infantil fue la de mayor interés.

**Conclusione:**

s: Este estudio demostró altos niveles de acuerdo entre los participantes sobre las prioridades de investigación en Covid-19 y post-Covid en Brasil.

**Ethical aspects**

This research respected ethical principles, having obtained the following approval data::

Research Ethics Committee: Universidade de Brasília

Opinion number: 54270821.6.0000.8093

Approval date: 23/6/2022

Certificate of Submission for Ethical Appraisal: 5.486.596

Informed Consent Form: Obtained from all participants prior to data collection.

## Introduction

The COVID-19 pandemic has had a direct impact on the socioeconomic conditions of global populations, leading to profound social and gender inequities in low- and middle-income countries (1-2). In Latin America, more than 30 million people fell below the poverty line after the start of the pandemic (3-4). The new virus has exposed the weaknesses of health systems and caused problems that require multifaceted health, environmental and socioeconomic policies (5).

In Brazil, COVID-19 has affected more than 37 million people. More than 700,000 deaths have been confirmed, in contexts of worsening poverty, inequality, and unemployment (6). The pandemic has highlighted the difficulties faced by the Brazilian National Health System (*Sistema Único de Saúde* - SUS), in a scenario of sharp reductions in funding and the weak structure of care networks, with restrictions on access to quality health facilities and prevalence of chronic noncommunicable conditions (7-9). Even following two critical years of the pandemic, Brazil is still dealing with significant mortality, long COVID-19 conditions, and their socioeconomic and social security effects on affected individuals and population groups.

The health science, technology and innovation system was fundamental in the response to the COVID-19 pandemic. Funders and researchers acted quickly and in unprecedented ways, defining research priorities and making significant investments, especially in high-income countries, to address the virus and mitigate its impacts on health systems (10-13).

Each government responded to the COVID-19 pandemic differently (11), influenced by available resources and funding (12). Prioritizing research was crucial for maximizing the impact of investments and strengthening national health systems (14–16,^5^). 

The World Health Organization led the way in defining global priorities and quickly coordinated strategies for immediate prioritization. It accelerated research that contributed to efforts to contain the spread of COVID-19 and provide health care for people affected by the disease, as well as the development of rapid diagnostics, drugs and vaccines to respond quickly to the pandemic (17). Some countries created their own research agendas (12-13).

Public policies and research policies are essential for creating lasting solutions focused on equality and social justice. The Ministry of Health and funding agencies in Brazil funded research during the pandemic, but there is still a need to deepen our understanding of COVID-19 and its consequences (18). 

This study proposed an agenda for COVID-19 and long COVID research priorities, in order to guide government and research funding agencies in optimizing health science, technology and innovation resources in Brazil.

## Methods

### Design 

This is a qualitative research project to develop an agenda for COVID-19 and long COVID-19 research priorities in Brazil, with the participation of social actors, developed between April 2022 and March 2023, conducted in two stages (19).

In the first stage, a wide consultation open to suggestions for COVID-19 research priorities was carried out between June and August 2022, organized according to the themes of the four axes of the COVID-19 Evidence Network to support Decision-making (COVID-END) initiative: I – Public-health measures; II – Clinical management of COVID-19 and pandemic-related conditions; III – Health system arrangements for COVID-19; and IV – Economic and social responses. 

COVID-END is an evidence network initiative aimed at supporting decision-making in the face of the pandemic (20). An electronic questionnaire was created for each axis, made available via Microsoft Google Forms and sent to the participants’ institutional emails, in which they indicated thematic lines for research priorities taking into account the themes of each axis. Each participant accessed the electronic questionnaire after signing the informed consent record.

In the second stage, a consensus analysis of priorities was carried out using the modified Delphi technique with a panel of experts in February and March 2023. This technique consists of rounds of semi-structured questionnaires based on the Likert scale, in which consensus is defined when the item obtains 70.0% approval. Its objective is to obtain a qualified collective opinion on certain issues from a group of selected experts (21). The thematic lines suggested in the broad consultation were analyzed, systematized according to related lines, transferred to the themes or axes for better adaptation or excluded in cases of repetition or inadequate formulation. Participants were individually invited, via email containing the access link to the electronic questionnaire, to respond in two rounds, after signing the consent record.

In the first round, participants evaluated the systematized research thematic lines using a Likert scale (I totally agree, I agree, I neither agree nor disagree, I disagree and I totally disagree). It was possible to list comments on each item. In the second round, after analyzing the consensus, a new electronic questionnaire was sent, containing the thematic research lines that did not achieve consensus in the first round and the new lines proposed by the panel of experts.

### Participants

In the first stage of the study, 1,028 participants were invited, 257 for each COVID-END axis, considering the five regions of Brazil, the participant’s activity profiles (researchers, health service and health science and technology managers, health professionals and heath service users) and gender (two men and two women for each profile). Of the total number of participants invited, 71 agreed to collaborate with the study, and three refused. The participants were selected through a survey of data in the public domain available on the internet (Table 1). 

**Table 1:** Distribution of participant profile by thematic axes of the COVID-19 Evidence Network to support Decision-making in the first and second stages of the study to define COVID-19 research priorities. Brazil, 2022-2023

First stage – wide consultation						
Axis	Researchers	Health service managers	Health science and technology managers	Health professionals	Health service users	Total
Public-health measures	7	5	0	5	4	21
Clinical management of COVID-19 and pandemic-related conditions	11	6	3	1	2	23
Health system arrangements for COVID-19	7	2	1	4	1	15
Economic and social responses	6	3	0	2	1	12
**Total**	31	16	4	12	8	71
Second stage – Delphi method						
Public-health measures	4	0	0	1	-	5
Clinical management of COVID-19 and pandemic-related conditions	2	1	0	3	-	6
Health system arrangements for COVID-19	2	0	3	0	-	5
Economic and social responses	1	0	2	1	-	4
**Total**	9	1	5	5	-	20

^a^The symbol (-) indicates that there were no participants.

The inclusion criteria for each of the profiles were as follows:

A. Researchers:

1. be registered on the Lattes Platform (Plataforma Lattes) – National Council for Scientific and Technological Development (CNPq);
2. have a productivity grant from the National Council for Scientific and Technological Development (CNPq);
3. have experience in three areas of knowledge about COVID-19 (health sciences, biological sciences and human sciences); and
4. have experience in the area of ​​COVID-19 accredited in postgraduate health sciences programs.

The exclusion criterion was not having a doctorate degree. 

B. Health service and health science and technology managers:

1. working in management of government agencies that promote science and technology in Brazil as a coordinator in state Health Departments and research support foundations;
2. working in management of government agencies such as Ministry of Health managers (Health Surveillance Secretariat, Department of Science and Technology); and
3. working in management of government bodies, such as municipal health service managers.

C. Health professionals:

1. working in management positions in the public university hospitals network – Rede Ebserh.

The exclusion criterion was not being employed in a health service during the pandemic. 

D. Health service users:

1. being a full member or alternate member representing health service users on formal bodies of social participation in the SUS (national and state health councils).

The sample for the second stage of the study was composed of a panel of experts including researchers, health service managers, health science and technology managers, and health professionals working in the area of ​​COVID-19. In this stage, the inclusion criteria for participants were: i) researchers with publications on COVID-19 in scientific journals; ii) managers who were working or had worked in the coordination of state Health Departments, research support foundations, and the Ministry of Health during the (post) pandemic period; and iii) health professionals who were working or had worked to address COVID-19.

In this study, considering possible losses, 28 participants were invited; 20 agreed to collaborate – the choice of the number was based on the literature that recommended an average of 15 participants (22-24). They were distributed between the four COVID-END axes to ensure analysis of the thematic lines. In turn, the Delphi technique is useful when the round involves a long questionnaire, grouping themes into specific areas so that the panel of members works on each area separately (25). The participants were not the same in stages 1 and 2. 

### Data analysis

For the qualitative analysis of the first stage of this study, the electronic questionnaires were exported to an Excel spreadsheet. Each proposal was analyzed individually and then categorized, grouped by similarity of content and responses were excluded that did not enable the theme to be understood, which generated the list of thematic lines distributed between the four axes and research themes. Word cloud analysis was performed using the Iramuteq application, with a minimum of three repetitions of words in the text.

In the statistical analysis of the questionnaires answered in the Delphi stage, the items were analyzed individually in each round. Consensus was defined when the item presented 70.0% positive approval (totally agree and agree). In this study, interquartile ranges were not presented because the sample size for each questionnaire was small. Positive approval was calculated using the following formula based on the Likert scale frequencies of each item among the interviewees:


$$Approval=\frac{\left(\sum \;agree)\;+\;(\sum \;\text{totally agree}\right)}{\left(\text{number of participants}\right)}\times \;100$$

Thematic lines that presented a consensus index lower than 70.0% were reformulated, according to the comments received by experts for the new Delphi round. 

## Results

In all, 91 participants contributed to the two stages of this study. In the first stage, 71 of them listed priority COVID-19 research topics, stratified into the four axes of the COVID-END initiative. Researchers accounted for the largest number of participants (43.6%), followed by health science and technology managers and health service managers (28.2%), health professionals (16.9%), and representatives of SUS users (11.3%) (Table 1). In the second stage, 20 and 18 experts participated in rounds 1 and 2 of the study. The profile of the experts remained close to that of stage 1, except for the absence of service user representatives (Table 1). In the wide consultation stage, 186 thematic lines of research on COVID-19 and long COVID were obtained, which were categorized according to the COVID-END initiative axes. After analysis, grouping by similarity, and exclusion of responses, a list of 161 thematic lines was generated, 42 of which were in Axis I – Public-health measures; 45 in Axis II – Clinical management of COVID-19 and pandemic-related conditions; 32 in Axis III – Health system arrangements for COVID-19; and 42 in Axis IV – Economic and social responses. 

Of the 161 thematic research lines evaluated by the panel of experts in round 1 of the Delphi method, 139 obtained a positive approval percentage equal to or greater than 70.0%. In that round, the experts proposed the inclusion of 51 more thematic lines of research. After analysis and systematization, these new proposals resulted in 41 new thematic lines, which were included in the electronic questionnaires to be evaluated in round 2.

In the second round, 63 thematic research lines were evaluated, resulting from the 22 that did not reach consensus, and 41 new lines that were added by the panel of experts in the first round. Of the 63 thematic lines examined, 40 achieved consensus (positive approval equal to or greater than 70.0%). As such, a set of 179 thematic lines of research priorities on COVID-19 and its effects was generated. The results of the consensus of the first and second rounds between the thematic lines of research in each axis of COVID-END were presented (Tables 4, 5, 6 and 7).

Fifty priority lines were listed in the three themes of Axis I – Public-health measures (Table 2). Most of the lines focused on the theme: “Broad spectrum public-health measures”, with 31 lines (62.0%). In the three themes, lines on preventive vaccines for COVID-19 were frequent. Of the 50 proposals evaluated, 76.0% achieved consensus in the first round.

**Table 2:** Result of the consensus of the first and second rounds of the Delphi method, classified according to the themes of Axis I – Public-health measures. Brazil, 2022-2023

Research themes	Research lines	First round^a^	Second round	No. of thematic lines
		Consensus (%)	Consensus(%)	
**Axis I**	**Public-health measures**	(n=5)	(n=5)	
**Prevention of infection**	Maintenance of annual COVID-19 vaccination.	100	-	12
	Strategies to increase vaccination coverage.	100	-	
	Evaluate the distribution of full vaccination according to demographic, geographic and socioeconomic indicators.	100	-	
	Impact of vaccination on incidence of the severe form of COVID-19.	100	-	
	Analysis of the causes of reduction in COVID-19 vaccination.	-	100	
	Quality and availability of access to vaccines for children and youth.	-	80	
	Analysis of access to vaccination and social determinants by gender and race.	-	100	
	Evaluation and monitoring of vaccination coverage in traditional communities and quilombolas.	-	100	
	Effectiveness and impact of adherence to COVID-19 prevention measures for different audiences.	100	-	
	Analysis of the COVID-19 prevention and communication strategies developed by social movements and research institutions.	100	-	
	Education interventions to control COVID-19.	100	-	
	Evaluation of the efficiency of health education and communication strategies in times of pandemics.	100	-	
**Infection control**	Evaluation of the dissemination of the positive effects of COVID-19 vaccination.	100	-	7
	Evaluation of the impact of COVID-19 using the Mortality Information System.	100	-	
	Evaluation of access to high cost COVID-19 medication.	60	80	
	Evaluation of access to and implementation of SARS-CoV-2 and genetic variant testing and follow-up programs in the health care network, urban and rural areas.	100	-	
	Development of practical and effective disinfection methods for the air in closed environments and for surfaces.	80	-	
	More effective behavioral measures (epidemiological and sociocultural) for COVID-19 infection control COVID-19.	100	-	
	Evaluation of behavior and lifestyle changes after the pandemic.	100	-	
**Broad spectrum public-health measures**	Evaluation of access to COVID-19 vaccination in Primary Health Care.	100	-	31
	Side effects of COVID-19 vaccines.	-	80	
	Evaluation of the Brazilian National Immunization Policy and its relationship with COVID-19 vaccination coverage.	100	-	
	Relevance of research and activities undertaken by public institutions arising from COVID-19.	80	-	
	Funding to promote research on COVID-19 prevention and surveillance.	80	-	
	Evaluation of funding from research promotion bodies, industries and donating institutions for research, development and innovation of essential supplies for COVID-19.	80	-	
	Evaluation of methodologies and interventions for training health professionals in prevention and control of COVID-19 and long COVID.	80	-	
	Impacts of COVID-19 on health professionals becoming ill and on their work processes health professionals.	100	-	
	Evaluation of information and prevention campaigns about COVID-19 on social media and other communication channels.	100	-	
	Evaluation of social participation in information campaigns about COVID-19.	100	-	
	Consequences of the infodemic and health communication for COVID-19.	80	-	
	Evaluation of the impact of COVID-19 on the population’s health.	100	-	
	Identification of groups more vulnerable to becoming severely ill and dying from COVID-19.	100	-	
	Analysis of COVID-19 morbidity and mortality among Black people.	-	100	
	Impacts of the COVID-19 pandemic on the health of children and adolescents.	80	-	
	Evaluation of the impact of COVID-19 on the psychosocial and mental health of the population.	100	-	
	Evaluation of the use of health services for COVID-19.	100	-	
	Analysis of the effects of COVID-19 on Amazon communities.	100	-	
	Mapping and monitoring of natural reservoirs of the SARS-CoV-2 family virus in the Neotropics and urban environments.	100	-	
	Side effects of COVID-19 vaccine on reduced visual acuity.	80	-	
	Effects of COVID-19 on pre-existing conditions in people with disabilities.	60	100	
	Long COVID effects in people with disabilities.	60	80	
	Implementation of a specialized care network for people with long COVID sequelae.	100	-	
	Ecological research on Primary Health Care-sensitive hospitalizations and COVID-19 and long COVID.	60	100	
	Evaluation of monitoring by Family Health and Specialized Care teams for the rehabilitation of long COVID cases.	100	-	
	Evaluation of incidences and sociodemographic characteristics of people with long COVID.	100	-	
	Long COVID in children and adolescents.	-	100	
	Biological and social risk factors associated with long COVID.	100	-	
	Assessment study on the burden of post-COVID syndrome in Brazil.	100	-	
	Use of rapid and available resources for the development of devices and equipment in the treatment of COVID-19.	80	-	
	Analysis of data disaggregated by race and color held on the vaccination information system.	-	100	

^a^In the First Round column, the symbol (-) indicates that the thematic research lines were assessed in the Second Round.

Forty-five thematic research lines were evaluated in Axis II – Clinical management of COVID-19 and pandemic-related conditions, of which 91.0% achieved consensus in the first round (Table 3). The themes “COVID-19 prophylaxis” and “Clinical management of pandemic-related impacts” stood out, with the largest number (10 lines, 22.0%). The proposals for these themes were more focused on vaccines and clinical support for hospitalized patients with COVID-19. With regard to the theme “COVID-19 management with a syndemic approach” (4 lines), most of the proposals were related to the impacts of the disease in Brazilian urban peripheral suburbs. As for the themes “Treatment for long COVID conditions” and “Clinical management of pandemic-related impacts” (8 and 10 lines), participants made proposals on physical, psychological, neurological and long COVID rehabilitation in immunosuppressed patients, infants and children. With regard to the theme “COVID-19 Health promotion” (4 lines), thematic lines were cited on the impact of physical exercise, mental health of health professionals and negative effects of the infodemic.

**Table 3:** Result of the consensus of the first and second rounds of the Delphi method, classified according to the themes of Axis II – Clinical management of COVID-19 and pandemic-related conditions. Brazil, 2022-2023

Research themes	Research lines	First round^a^	Second round	No. of thematic lines
		Consensus (%)	Consensus (%)	
**Axis II**	**Clinical management of COVID-19 and pandemic-related conditions**	(n=6)	(n=6)	
**COVID-19 prophylaxis**	Updating and development of preventive and therapeutic vaccines with new technologies for viral variants.	100	-	10
	Immune response to the vaccine in risk groups.	100	-	
	Evaluation of vaccine response in children and youth.	-	100	
	Development of multicenter phase III clinical trials for new vaccines.	100	-	
	Strategies for expanding the number of doses of COVID-19 immunization vaccines.	83	-	
	Availability of immunobiologicals for children and youth with chronic diseases.	-	83	
	Development of inputs for vaccine manufacturing in Brazil.	100	-	
	Development of diagnostic inputs for COVID-19 and other endemic and epidemic diseases in Brazil.	100	-	
	Development of new drugs for COVID-19 and post-SARS-CoV-2 exposure.	83	-	
	Development of drugs to extend protection against COVID-19 for immunosuppressed patients.	83	-	
**Clinical treatment of COVID**-19	Retrospective cohort studies to evaluate the effectiveness of corticosteroid and antiviral treatments for COVID-19.	83	-	9
	Evaluation of adverse events due to vaccines.	-	100	
	Use of antiviral drugs - Paxlovid (nirmatrelvir + ritonavir) and molnupiravir for patients with COVID-19 in outpatient settings.	83	-	
	Effectiveness of COVID-19 treatment for immunosuppressed individuals and at-risk groups.	100	-	
	Implementation and evaluation of clinical protocols and therapeutic guidelines for home, hospital (ICUs and post-intensive care syndrome) and long COVID-19 treatment.	100	-	
	Systematic reviews of the effectiveness of COVID-19 treatment strategies.	100	-	
	Safe care mechanisms for asymptomatic patients: risks and care protocols.	83	-	
	Research on supportive care for patients with neurological COVID-19 symptoms.	83	-	
	Qualitative study on post-discharge health service care for people hospitalized with COVID-19 in intensive care units.	83	-	
**COVID-19 management with a syndemic approach**	Studies on surveillance of SARS-CoV-2 deaths with emphasis on defining criteria for classification and integration between reporting and mortality information systems.	100	-	4
	Mental health care in Brazilian peripheral suburbs in the context of COVID-19.	100	-	
	Organization and quality of care in Brazilian peripheral suburbs in the context of COVID-19 and long COVID.	100	-	
	Studies on interrelationships between COVID-19 and other endemic infectious diseases in the long term.	100	-	
**Treatment for long COVID conditions**	Effectiveness of practices for the physical and mental rehabilitation of long COVID patients.	100	-	8
	Effects and psychological needs of patients with COVID-19.	100	-	
	Understanding neurological manifestations and clinical management of long COVID.	100	-	
	Monitoring the progression of specific COVID-19 sequelae (dyspnea, memory deficit, insomnia, depression).	100	-	
	Prospective evaluation of people recovered from COVID-19 with a clinical-laboratory approach.	83	-	
	Effectiveness of long COVID treatment for immunosuppressed individuals and at-risk groups.	100	-	
	Sequelae and effects of long COVID in babies and children.	100	-	
	Diagnosis of the specialized network in Brazilian municipalities for the care of conditions due to hospitalization in intensive care units and long COVID.	83	-	
**Clinical management of pandemic-related impacts**	Survey on clinical management adopted in Brazilian states for COVID-19.	83	-	10
	Evaluation of care flows during the COVID-19 pandemic.	100	-	
	Evaluation of access to clinical follow-up of COVID-19 sequalae.	83	-	
	Best psychological intervention practices to address the psychosocial consequences of COVID-19.	100	-	
	Evaluate efficient clinical management for patients with pulmonary COVID-19 sequelae.	100	-	
	Studies on clinical management of immune deficiencies and cancer and interrelationship with COVID-19 infections and reinfections.	83	-	
	Impact of the COVID-19 pandemic on incidence and mortality from different causes.	100	-	
	Impact of the COVID-19 pandemic on diagnosis, monitoring and treatment for different causes.	100	-	
	Impact of the COVID-19 pandemic on birth rates and sexual and reproductive health.	83	-	
	Health conditions neglected because of the COVID-19 pandemic.	83	-	
**COVID-19 health promotion**	Identification of the best care interventions for maintaining health.	67	100	4
	Impact of physical exercise as a measure to promote health and prevent COVID-19.	100	-	
	Observational studies associated with the mental health of healthcare professionals with and without COVID-19 patient workloads.	83	-	
	Negative effects of the infodemic on COVID-19 health promotion.	50	83	

^a^In the First Round column, the symbol (-) indicates that the thematic research lines were assessed in the Second Round.

Axis III – Health system arrangements for covid-19, with 35 lines, had the largest number of proposals in the themes “Organization of service provision for COVID-19” (11 lines, 31.0%) and “Governance arrangements for COVID-19” (10 lines, 29.0%) (Table 4). In the first three themes “Cross-cutting arrangements that need a systemic approach to COVID-19”, “Organization of service provision for COVID-19” and “Financial arrangements for COVID-19”, the study participants’ attention was focused on topics related to mental health, children and immunosuppressed individuals, organization and transfer of financial resources at the three levels of health care. In “Governance arrangements for COVID-19” (who can make which decisions), topics related to the federal government’s response through decision-making for the COVID-19 pandemic prevailed. In this axis, 71.0% of the thematic lines achieved consensus in the first round. 

**Table 4:** Result of the consensus of the first and second rounds of the Delphi method, classified according to the themes of Axis III – Health system arrangements for COVID-19. Brazil, 2022-2023

Research themes	Research lines	First round^a^	Second round	No. of thematic lines
		Consensus (%)	Consensus (%)	
**Axis III**	**Health system arrangements for COVID**-19	(n=5)	(n=5)	
**Cross-cutting arrangements that need a systemic approach to COVID**-19	Systemic approach to COVID-19, comorbidities and eating, nutrition and physical activity habits.	80	-	7
	Systemic approach to COVID-19 and mental health.	100	-	
	Systemic approach para COVID-19 and children.	-	80	
	Systemic approach para COVID-19 and immunosuppressed individuals+.	-	80	
	Evaluation of the mandatory reporting system for cases of influenza-like illness.	-	80	
	Online mental health intervention models for long COVID patients.	100	-	
	Analysis of the preparedness, response and resilience capacity of the Brazilian health system to public health emergencies.	100	-	
**Organization of service provision for COVID**-19	Organization of the healthcare network (primary, medium and high complexity) for long COVID.	100	-	11
	Impacts of COVID-19 on the use of outpatient, hospital and Primary Health Care services for different causes.	100	-	
	Evaluation of the adequacy of health network infrastructure for COVID-19.	80	-	
	Planning the demand for primary, secondary and tertiary health services to address COVID-19 at the three levels of government.	100	-	
	Evaluation of government services for the provision of hospital beds and essential supplies at the three levels of health care, in the fight against COVID-19.	100	-	
	Evaluation of field hospitals created during the COVID-19 pandemic.	-	80	
	Impacts of COVID-19 on Primary Health Care structure and organization.	60	100	
	Organization of the private and supplementary health service network for COVID-19.	80	-	
	Evaluation of the use of health facilities for long COVID patients and vulnerable populations.	60	80	
	Impacts of COVID-19 on non-COVID-19 patients on waiting lists and morbidity and mortality for elective surgeries in different specialties.	100	-	
	Evaluation of the results of mortality and case fatality ratio due to COVID-19 in the different care models implemented in municipalities in the public system.	80	-	
**Financial arrangements for COVID**-19	Cost-benefit evaluation for the acquisition and use of rapid antigen tests for mild cases of COVID-19.	-	80	7
	Evaluation of the impact of federal financial resource transfers to states and municipalities in response to the COVID-19 pandemic.	100	-	
	Financial impacts of COVID-19 on public health.	100	-	
	Financial resources and trained professionals for rehabilitation and social reintegration of people with long COVID.	80	-	
	Financial incentives to expand access to specialized services for patients with COVID-19 post-intensive care syndrome in the Brazilian municipalities.	100	-	
	Vulnerable population access to COVID-19 drugs approved by the World Health Organization.	60	100	
	Cost-effectiveness of treating COVID-19 and long COVID COVID.	80	-	
**Governance arrangements for COVID-19** (**who can make which decisions**)	Case studies of the response of state and municipal health departments to COVID-19.	100	-	10
	State intervention in private life in times of COVID-19.	80	-	
	Use of World Health Organization recommendations by the federal government.	80	-	
	Comparative studies between federal and municipal governments for COVID-19 control measures in urban areas.	60	80	
	Federative pact and National Immunization Program.	100	-	
	Benchmarking study of COVID-19 governance practices in other countries.	80	-	
	Participation of technical chambers and committees as spaces for building consensus and decisions in the COVID-19 health crisis.	80	-	
	Evaluation of the participation of experts and health councils in decision-making during and after COVID-19 outbreaks.	80	-	
	Health Crisis and Social Security.	80	-	
	Impacts and influence of the federal government on the organization of the SUS and the use of precautions to prevent and address COVID-19.	60	80	

^a^In the First Round column, the symbol (-) indicates that the thematic research lines were assessed in the Second Round.

In Axis IV – Economic and social responses, 49 research priorities were listed. The most prominent themes were education (8 lines, 16.0%) and work (7 lines, 14.0%). Within these themes, the largest number of thematic lines indicated by respondents refer to medicines, health services, health system governance, education and work related to COVID-19 (Table 5).

**Table 5:** Result of the consensus of the first and second rounds of the Delphi method, classified according to the themes of Axis IV – Economic and social responses. Brazil, 2022-2023

Research themes	Research lines	First round^a^	Second round	No. of thematic lines
		Consensus (%)	Consensus (%)	
**Axis IV**	**Economic and social responses**	(n=4)	(n=3)	
**Services for children and youth**	Consequences of social isolation for newborns and children under five years of age in the COVID-19 pandemic.	100	-	2
	Child and youth sociability and mental health in the post-COVID-19 pandemic context.	100	-	
Citizenship	Impact of culture and citizenship in addressing COVID-19.	100	-	2
	Provision and evolution of public services in the COVID-19 pandemic.	100	-	
**Social and community services**	Effects of COVID-19 on the provision of social and community services for people in vulnerable situations.	100	-	5
	Participation of civil society organizations in the fight against COVID-19.	100	-	
	Evaluation of biopsychosocial support projects run by the third sector and innovative methods for their dissemination.	100	-	
	Access to information about COVID-19 by residents of peripheral suburbs.	-	100	
	Availability and supply of hygiene kits (mask, alcohol gel) for peripheral communities.	-	100	
**Culture and gender**	Impact of COVID-19 on the division of work and health between the sexes.	75	-	3
	Development and dissemination of innovative and online cultural activities suitable for different age groups in the post-COVID-19 pandemic period.	75	-	
	Impact of COVID-19 on gender inequalities.	75	-	
**Development and economic growth**	Civil society organization contributions to the development and economic growth of vulnerable communities to protect against COVID-19.	100	-	3
	The role of the State in private companies and commerce in the COVID-19 pandemic.	100	-	
	Economic and social responses to the consequences of discouragement and delay in the widespread vaccination of the Brazilian population against COVID-19.	100	-	
Education	Evaluation of the impact of the COVID-19 pandemic on learning in public elementary and high schools in the Brazilian municipalities.	100	-	8
	Development of innovative distance learning methods for primary, secondary and higher education.	100	-	
	Impact of remote learning technologies on teaching.	-	100	
	School dropout and repetition in times of COVID-19.	-	100	
	Evaluation of teacher unemployment during the pandemic and post-pandemic period.	-	100	
	Civil society organization contributions in the development of platforms for education programs and projects.	100	-	
	Impacts of COVID-19 on teaching work in primary, secondary and higher education and mental health.	100	-	
	Interventions to address losses caused by the COVID-19 health crisis in public education.	100	-	
Work	Loss of vital function and work related to COVID-19.	100	-	7
	Suicidal ideation and suicide among agribusiness workers during the COVID-19 pandemic.	100	-	
	Analysis of job offers before and after the COVID-19 pandemic.	-	100	
	Impact of COVID-19 on the domestic workers and other general service professionals segment.	-	100	
	Impact of the COVID-19 health crisis on Brazil’s employment and income policies.	100	-	
	Analyze the effect of COVID-19 on the formal and informal labor market in different sectors of the economy.	100	-	
	Studies on social vulnerability and the impact of the absence of mitigation measures in the world of work and education.	100	-	
**Financial protection**	Evaluation of income transfer and minimum income proposals for people affected in their work activity by the pandemic.	100	-	6
	Evaluation of coverage and targeting of government financial protection programs to compensate for income losses due to COVID-19.	75	-	
	Economic and social support needs for children and youth with family losses due to COVID-19 and people with long COVID.	100	-	
	COVID-19 and the impact of minimum income policies on the social determinants of health.	75	-	
	Evaluation of the impact of COVID-19 consequences on the income of families in situations of social vulnerability.	100	-	
	Impact of COVID-19 mortality on the economically active population.	100	-	
**Food security and safe food**	Municipal, state and national food security before and during the COVID-19 pandemic.	100	-	3
	Evaluation of coverage and targeting of basic food basket and ready-made meal distribution programs resulting from family income losses.	100	-	
	Guaranteeing safe food for people with incomes below the minimum wage.	100	-	
Housing	Development of innovative architectural projects to ensure adequate ventilation in individual housing (houses) and collective housing (apartment buildings).	50	100	4
	Housing alternatives for the homeless population.	-	100	
	Housing and COVID-19: effects of precarious housing conditions and the spread of COVID-19.	-	100	
	Impacts of government and social projects to promote social housing.	-	100	
Infrastructure	Analysis of urban conditions and the spread of the SARS-CoV-2 virus.	100	-	1
Transport	Public transport crowding and risks of COVID-19 contamination and infection.	100	-	
	Development of practical and effective methods for disinfecting public transport vehicles.	100	-	
	Impact on public transport planning or model in the post-COVID-19 pandemic period.	100	-	5
	Public transport and COVID-19: actions by municipal, state and federal governments and private companies to contain its spread.	-	100	
	Impact on the transport sector during and after the COVID-19 pandemic.	-	100	

^a^In the First Round column, the symbol (-) indicates that the thematic research lines were assessed in the Second Round.

The similarity of terms in the themes verified through word cloud analysis showed that research lines related to impact and evaluation were a cross-cutting concern in the four axes among the participants in this study. Just as long COVID appears in the four COVID-END axes, we also found that it was a research priority among the participants in this study.

In the analysis as a whole, mental illnesses and immunosuppression situations stood out, identified in Axis II – Clinical management of COVID-19 and pandemic-related conditions. The research lines on mental illnesses were related to the effects on Brazilian peripheral suburbs, effects on the rehabilitation of long COVID patients and on health professionals. Illnesses in immunosuppression situations are related to research on the effectiveness of treatment during long COVID.

Considering the repetition of words in three of the four axes, children were the priority population among the study participants. The priority lines related to this group were related to long COVID, quality and supply of vaccines, vaccine response, immunobiologicals for chronic diseases, consequences of social isolation, sociability in the post-pandemic context and economic support for children with family losses resulting from COVID-19.

## Discussion

This study identified COVID-19 and long COVID-19 research priorities from the perspective of social actors in areas of knowledge that include clinical conditions, magnitude and social determinants, and social and economic impacts of COVID-19. Among the research lines listed by consensus among the participants, studies on evaluation, social and health impacts of covid-19, long COVID, and their sequelae predominated. Mental illnesses and immunosuppression stood out among the set of illnesses. Children were the population of greatest interest among the participants. Some of the priority research topics pointed to studies on medications, health service responses, health system governance, and the effects of COVID-19 on education and work.

The limitations of the study relate to low adherence and lack of diversity in the profile of the participants, especially SUS managers and service users. The themes presented may not cover the impacts of COVID-19 and its consequences on the Brazilian population widely, since the pandemic affected social groups in different ways. This study was completed within a period of one year, in which the magnitude of the COVID-19 pandemic changed in Brazil and new interventions were needed to address its consequences. The use of priority research themes should consider these changes and the need to perform prioritization ranking in the short, medium and long term.

COVID-19 has had a number of impacts on various areas of society. In healthcare services, the constant increase in cases of the disease between 2020 and 2021 caused collapses in healthcare and research systems around the world, forcing large-scale reorganization of hospitals and improvements in infrastructure and financial resources in the SUS (26).

The pandemic has affected education by changing the learning process in schools. Brazil was one of the countries that spent the longest time with schools closed due to social isolation and had remote learning as an alternative (27). With regard to the Brazilian labor market, it was found that, in the first months of the pandemic, unemployment rates increased, and workers migrated from formal to informal jobs (28). The impacts of COVID-19 have proven to be relevant at the national and international levels for COVID-19 research priority agendas. The study of COVID-19 research priorities in Southeast Asia has also been presented as a theme (5,11-13).

Clinical manifestations of the virus and recurrent or persistent symptoms after infection have been termed “post COVID-19 conditions” or “long COVID” (29). In 2021, of the 1,790,796 deaths recorded in Brazil, 2,948 were due to long COVID causes, approximately 1.6 deaths due to long COVID-19 for every 1,000 records. Between March and April 2022, of the 720 individuals who tested positive for covid-19, 69.0% had symptoms for more than three months with effect from the onset of acute illness (30-31).

Mental health has been highlighted as a research priority due to the psychological and social impacts of the pandemic, which soon became evident in the first weeks of the pandemic (32). There is concern about the impact of COVID-19 on at-risk populations, especially immunocompromised individuals, who are more vulnerable to infections and serious complications (33). It is essential to focus research development on these areas.

Although the risk of severe COVID-19 cases and mortality is lower among children, they can develop critical conditions. The lack of inclusion of this population in the initial development of treatments is worrying (34-35). In addition to becoming ill, the implications of social isolation for children have caused the abrupt interruption of most face-to-face human interactions, causing excessive use of the internet, often without parental or guardian supervision, which has placed children and adolescents in a variety of vulnerable situations (36). Although there are studies on the impact of the pandemic on children, there are still considerable knowledge gaps on this topic (12-13). It is essential to ensure that research on health, education, and sociability is prioritized to develop actions and strategies for this population.

COVID-19 and long COVID impact health, life and social interaction, highlighting the need for research into solutions. A priority agenda guides investments, monitors results and updates actions according to the health situation.

Using the four axes of the COVID-END initiative in formulating the questionnaires was a strong point of the study, facilitating an online and comprehensive approach with a diversity of participants. The initiative was focused on decision-making about COVID-19 and addressed topics such as prevention, clinical management, organization of health systems and economic and social responses, being one of the 50 main global evidence synthesis groups (20).

Another point to be highlighted was the use of the methodology that was consolidated in two stages: i) the broad consultation that enabled identification of priority research lines, based on the composition and selection process of researchers from different areas of knowledge, health service managers and health professionals with direct experience and knowledge in management and assistance during and after the pandemic, health science and technology managers who launched calls for proposals on COVID-19 and SUS health service users; and ii) definition of consensus on prioritization using the Delphi method with a panel of COVID-19 experts.

Defining COVID-19 and post-COVID-19 research priorities without prior restriction on research type facilitates funding for diverse approaches. This ensures that policies and programs are evidence-based, filling knowledge gaps, preventing future crises, and promoting multidisciplinary and complementary research (5,37).

The results are important for countries like Brazil, which have limited resources for research, and it is essential to allocate these resources efficiently. Prioritizing research during the pandemic has been adopted by several countries, such as Iran and nations in Asia and Latin America, aligning priorities with local needs (5,38-39).

Effective scientific production in epidemics can be achieved with multidisciplinary research structures, regulation, sustainable funding, partnerships and trust, continuing to take place in the periods between epidemics (10). It is important to emphasize that this is not a closed agenda and nor should it be, as it requires continuous efforts and permanent updating according to the health and epidemiological context of COVID-19 and the consequences of long COVID.

Funding research aligned with prioritization is an efficient strategy for strengthening science and technology, in addition to helping in the formulation of public policies, especially for vulnerable populations, who are the most affected in emergencies.

## Data Availability

The database and analysis codes used in this research are available at: https://docs.google.com/spreadsheets/d/1C2hU3oqWFJe8VSiLpdhVfpVT82yhlQrQ/edit?gid=1623398596#gid=1623398596
