# Supplementary material for: Agenda for COVID-19 and long COVID research priorities in Brazil: results of wide consultation and Delphi consensus, 2022-2023
Source: Epidemiol Serv Saude. 2025 Jun 20;34:e20240623. doi: 10.1590/S2237-96222025v34e20240623.en (PMC12208623; doi:10.1590/S2237-96222025v34e20240623.en)
Supplement: Translation

# Translation: Agenda de prioridades de pesquisa em covid-19 e pós-covid no Brasil: resultados de consulta ampla e consenso Delphi, 2022-2023

**DOI:** 10.1590/S2237-96222025v34e20240623.pt

**Authors:** Natália Silva Alves, Everton Nunes da Silva, Gabriela Bardelini Tavares Melo, Micaela Alexandra Silva Paulino, Antonia Angulo-Tuesta

**Affiliations:** 1 Universidade de Brasília, Faculdade de Ciências e Tecnologias em Saúde, Programa de Pós-Graduação em Ciências e Tecnologias em Saúde, Brasília, DF, Brasil; 2 Ministério da Saúde, Secretaria de Ciência, Tecnologia e Inovação do Complexo Econômico Industrial da Saúde, Departamento de Ciência e Tecnologia, Brasília, DF, Brasil; 3 Universidade de Brasília, Faculdade de Ciências da Saúde, Programa de Pós-Graduação em Saúde Coletiva, Brasília, DF, Brasil

**Keywords:** Agenda de Pesquisa em Saúde, Síndrome de Covid-19 Pós-Aguda, Covid-19, Brasil, Estudo Qualitativo Exploratório

## Abstract

**Objetivo:**

Propor agenda de prioridades de pesquisa sobre covid-19 e pós-covid, a fim de orientar órgãos governamentais e de fomento à pesquisa para a otimização de recursos em ciência, tecnologia e inovação em saúde no Brasil.

**Métodos:**

Trata-se de estudo qualitativo, realizado em duas etapas, entre abril de 2022 e março de 2023. Na primeira etapa, realizou-se consulta ampla para a identificação de prioridades de pesquisa segundo os eixos da iniciativa COVID-19 Evidence Network to support Decision-making, com 71 participantes entre pesquisadores, gestores de saúde e de ciência e tecnologia, profissionais e usuários dos serviços de saúde. Na segunda etapa, foi realizado o consenso das prioridades propostas na etapa anterior, usando o método Delphi, com painel de 20 especialistas em covid-19 na primeira rodada e de 18 na segunda rodada.

**Resultados:**

Na consulta ampla, foram recebidas 186 linhas prioritárias de pesquisa sobre covid-19 e consolidadas em 161 linhas. Destas, 139 obtiveram o consenso na primeira rodada do método Delphi, e mais 40 linhas foram recebidas e incluídas na segunda rodada para consenso. A agenda proposta tem 179 linhas de pesquisa em covid-19. Predominaram temas de avaliação, impacto e sequelas da covid-19, covid-19 longa, doenças mentais e imunossupressão. A população de crianças foi de maior interesse.

**Conclusões:**

Este estudo demonstrou altos níveis de concordância entre os participantes sobre as prioridades de pesquisa em covid-19 e pós-covid no Brasil.

**Aspectos éticos**

A presente pesquisa respeitou os princípios éticos, obtendo os seguintes dados de aprovação: :

Comitê de ética em pesquisa: Universidade de Brasília

Número do parecer: 54270821.6.0000.8093

Data de aprovação: 14/4/2023

Certificado de apresentação de apreciação ética: 5.486.596

Registro do consentimento livre e esclarecido: Obtido de todos os participantes antes da coleta.

## Introdução

A pandemia causada pela covid-19 impactou diretamente as condições socioeconômicas das populações globais, provocando profundas iniquidades sociais e de gênero nos países de baixos e médios ingressos (1-2). Na América Latina, mais de 30 milhões de pessoas ficaram abaixo da linha da pobreza após o início da pandemia (3-4). O novo vírus desvelou as fragilidades dos sistemas de saúde e provocou problemas que necessitam de políticas multifacetadas de saúde, ambientais e socioeconômicas (5). 

No Brasil, a covid-19 acometeu mais de 37 milhões de pessoas. Foram confirmados mais de 700 mil óbitos, em contextos de recrudescimento da pobreza, desigualdades e desemprego (6). A pandemia mostrou as dificuldades que enfrentava o Sistema Único de Saúde (SUS), em um cenário de forte redução do financiamento e da débil estrutura das redes de atenção com restrições ao acesso a equipamentos de saúde de qualidade e à prevalência de condições crônicas não transmissíveis (7-9). Mesmo após os dois anos críticos da pandemia, o país ainda lida com a considerável mortalidade, as condições pós-covid-19 e seus efeitos socioeconômicos e de seguridade social nos indivíduos afetados e nos grupos populacionais. 

O sistema de ciência, tecnologia e inovação em saúde foi fundamental na resposta à pandemia de covid-19. Financiadores e pesquisadores atuaram rapidamente e de forma inédita, definindo prioridades de pesquisa e realizando investimentos significativos, especialmente em países de alta renda, para enfrentar o vírus e mitigar seus impactos nos sistemas de saúde (10-13). 

Cada governo respondeu à pandemia de covid-19 de forma distinta (11), influenciado por recursos e financiamento disponíveis (12). A priorização da pesquisa foi crucial para maximizar o impacto dos investimentos e fortalecer os sistemas nacionais de saúde (14-16,5). 

A Organização Mundial da Saúde liderou a definição de prioridades globais e coordenou rapidamente estratégias de priorização imediata. Acelerou a realização de pesquisas que contribuíram com ações de contenção da propagação da covid-19 e nos cuidados em saúde para pessoas acometidas pela doença, assim como o desenvolvimento de diagnósticos rápidos, medicamentos e vacinas para responder de forma ágil à pandemia (17). Outros países criaram suas próprias agendas de pesquisa (12-13).

As políticas públicas e de pesquisa são essenciais para criar soluções duradouras com foco na igualdade e justiça social. O Ministério da Saúde e as agências de fomento no Brasil financiaram pesquisas durante a pandemia, mas ainda deve-se aprofundar a compreensão da covid-19 e suas consequências (18). 

Este estudo propôs agenda de prioridades de pesquisas para covid-19 e pós-covid, a fim de orientar órgãos governamentais e de fomento à pesquisa para a otimização de recursos em ciência, tecnologia e inovação em saúde no Brasil.

## Método

### Desenho do estudo 

Trata-se de pesquisa qualitativa para a elaboração de agenda de prioridades de pesquisa em covid-19 e pós-covid-19 no Brasil, com a participação de atores sociais, desenvolvida entre abril de 2022 e março de 2023, conduzida em duas etapas (19).

Na primeira etapa, foi realizada consulta ampla de sugestões abertas de prioridades de pesquisa em covid-19, entre junho e agosto de 2022, organizada de acordo com os temas dos quatro eixos da iniciativa COVID-19 Evidence Network to support Decision-making (COVID-END): I – Medidas de saúde pública; II – Manejo clínico de covid-19 e seus impactos relacionados à pandemia; III – Organizações dos sistemas de saúde; e IV – Respostas econômicas e sociais. 

A COVID-END trata-se de iniciativa de rede de evidências, com intuito de apoiar a tomada de decisão diante da pandemia (20). Para cada eixo, foi elaborado um questionário eletrônico disponibilizado pelo Microsoft Google Forms e enviado para o e-mail institucional dos(as) participantes, no qual indicaram linhas temáticas de prioridades de pesquisa tendo em conta os temas de cada eixo. O participante acessava o questionário eletrônico após assinar o registro do consentimento livre e esclarecido.

Na segunda etapa, foi realizada a análise de consenso de prioridades utilizando-se a técnica Delphi modificada, em fevereiro e março de 2023, com um painel de especialistas. Essa técnica consiste em rodadas de questionários semiestruturados com base na escala Likert, na qual se define consenso quando o item obtém 70,0% de aprovação. Seu objetivo é a obtenção da opinião coletiva qualificada sobre determinadas questões de um grupo de especialistas selecionados (21). As linhas temáticas levantadas na consulta ampla foram analisadas, sistematizadas entre aquelas relacionadas, transferidas para os temas ou eixos para melhor adequação ou excluídas em casos de repetição ou formulação inadequada. Os(as) participantes foram convidados(as) individualmente, via e-mail com o link de acesso ao questionário eletrônico, após assinatura do registro de consentimento, a responderem em duas rodadas. 

Na primeira rodada, os(as) participantes avaliaram as linhas temáticas de pesquisa sistematizadas, através da escala Likert (concordo totalmente, concordo, nem concordo e não discordo, discordo e discordo totalmente). Foi possível elencar comentários em cada item. Na segunda rodada, após a análise do consenso, foi enviado novo questionário eletrônico, contendo as linhas de pesquisa temáticas que não obtiveram consenso na primeira rodada e as novas linhas propostas pelo painel de especialistas. 

### Participantes 

Na amostra da primeira etapa do estudo, foram convidados 1.028 participantes, 257 para cada eixo da COVID-END, considerando as cinco regiões brasileiras, os perfis de atuação (pesquisadores/as, gestores/as de saúde e de ciência e tecnologia, profissionais de saúde e usuários) e o sexo (dois homens e duas mulheres por cada perfil). Do total de convidados(as), 71 aceitaram colaborar com o estudo, e três recusaram-se. A seleção dos participantes ocorreu por meio do levantamento de dados de domínio público e disponíveis na internet (Tabela 1).

**Tabela 1:** Distribuição do perfil de participantes por eixos temáticos da COVID-19 Evidence Network to support Decision-making nas primeira e segunda etapas do estudo de definição de prioridades de pesquisa em covid-19. Brasil, 2022-2023

Primeira etapa – consulta ampla						
Eixo	Pesquisador(a)	Gestor(a) de saúde	Gestor(a) de ciência e tecnologia	Profissionais da saúde	Usuários(as)	Total
Medidas de saúde pública	7	5	0	5	4	21
Manejo clínico de covid-19 e questões de saúde relacionadas à pandemia	11	6	3	1	2	23
Organização dos sistemas de saúde para a covid-19	7	2	1	4	1	15
Respostas econômicas e sociais	6	3	0	2	1	12
**Total**	31	16	4	12	8	71
Segunda etapa – método Delphi						
Medidas de saúde pública	4	0	0	1	-^a^	5
Manejo clínico de covid-19 e questões de saúde relacionadas à pandemia	2	1	0	3	-^a^	6
Organização dos sistemas de saúde para a covid-19	2	0	3	0	-^a^	5
Respostas econômicas e sociais	1	0	2	1	-^a^	4
**Total**	9	1	5	5	-^a^	20

^a^O símbolo (-) indica que não houve participantes.

Os critérios de inclusão de cada um dos perfis foram os elencados a seguir. 

A. Pesquisadores(as):

1. estar cadastrado(a) na Plataforma Lattes – Conselho Nacional de Desenvolvimento Científico e Tecnológico;
2. possuir bolsa de produtividade do Conselho Nacional de Desenvolvimento Científico e Tecnológico;
3. ter experiência em três áreas do conhecimento sobre covid-19 (ciências da saúde, ciências biológicas e ciências humanas); e
4. ter experiência na área de covid-19 credenciados nos programas de pós-graduação em ciências da saúde.

O critério de exclusão foi não possuir título de doutor(a). 

B. Gestores(as) de saúde e de ciência e tecnologia:

1. atuar na gestão dos órgãos governamentais de fomento de ciência e tecnologia no Brasil como coordenador(a) nas secretarias estaduais de saúde e nas fundações de amparo à pesquisa;
2. atuar na gestão dos órgãos governamentais tais como gestores(as) do Ministério da Saúde (Secretaria de Vigilância em Saúde, Departamento de Ciência e Tecnologia); e
3. atuar na gestão dos órgãos governamentais tais como gestores(as) municipais de saúde.

C. Profissionais da saúde:

1. atuar nos cargos de direção da rede de hospitais universitários públicos – Rede Ebserh.

O critério de exclusão foi não estar vinculado(a) a uma unidade de saúde no período da pandemia. 

D. Usuários(as):

1. ser membro(a) titular e suplente da representação de usuários em instâncias formais de participação social no SUS (conselhos nacional e estaduais de saúde).

A amostra da segunda etapa do estudo foi composta por painel de especialistas entre pesquisadores(as), gestores(as) de saúde e de gestores(as) de ciência e tecnologia e profissionais da saúde com atuação na área de covid-19. Nesta etapa, os critérios de inclusão dos(as) participantes foram: i) pesquisadores(as) com publicações sobre covid-19 em periódicos científicos; ii) gestores(as) atuantes ou que atuaram nas coordenações das secretarias estaduais de saúde, das fundações de amparo à pesquisa e do Ministério da Saúde no período (pós) pandêmico; e iii) profissionais de saúde que atuam ou atuaram diante da covid-19.

Neste estudo, considerando-se as possíveis perdas, foram convidados(as) 28 participantes; 20 aceitaram colaborar – a escolha do número fundamentou-se na literatura que recomendava em média 15 participantes (22-24). Eles(as) foram distribuídos(as) entre os quatro eixos da COVID-END para garantir a análise das linhas temáticas. Na técnica Delphi, é útil quando a rodada for um questionário longo, agrupando-se temas em áreas específicas para que o painel de membros trabalhe em cada área separadamente (25). Nas etapas 1 e 2, os(as) participantes foram distintos(as). 

### Análise dos dados

Para a análise qualitativa da primeira etapa deste estudo, os questionários eletrônicos foram exportados para planilha Excel. Cada proposta foi analisada individualmente e, em seguida, foi realizada a categorização, o agrupamento por semelhança de conteúdo e a exclusão de respostas que não permitiam o entendimento do tema, o que gerou a lista de linhas temáticas distribuídas entre os quatro eixos e temas de pesquisa. Foi realizada a análise de nuvem de palavras, utilizando-se o software Iramuteq, com o mínimo de três repetições de palavras no texto.

Na análise estatística dos questionários aplicados na etapa Delphi, os itens foram analisados individualmente em cada rodada. O consenso foi definido quando o item apresentou 70,0% de aprovação positiva (concordo totalmente e concordo). Neste estudo, não foram apresentados os intervalos interquartis por se tratar de amostra pequena para cada questionário. A aprovação positiva foi calculada pela seguinte fórmula com base nas frequências da escala Likert de cada item entre os entrevistados:


$$Aprovação\;=\frac{\left(\sum \;concordo)\;+\;(\sum \;\text{concordo totalmente}\right)}{\left(\text{número de participantes}\right)}\times \;100$$

As linhas temáticas que apresentaram índice de consenso inferior a 70,0% foram reformuladas, de acordo com os comentários recebidos pelos especialistas para nova rodada Delphi. 

## Resultados

Nas duas etapas deste estudo, contribuíram 91 participantes. Na primeira etapa, 71 elencaram temas prioritários de pesquisa sobre covid-19, estratificados nos quatro eixos da iniciativa COVID-END. A maior participação foi de pesquisadores(as) (43,6%) seguida de gestores de ciência e tecnologia e de saúde (28,2%), profissionais da saúde (16,9%) e representantes de usuários do SUS (11,3%) (Tabela 1). Na segunda etapa, 20 e 18 especialistas participaram nas rodadas 1 e 2 do estudo. O perfil dos especialistas manteve-se próximo ao da etapa 1, exceto pela ausência de representantes dos usuários (Tabela 1). Na etapa de consulta ampla, obtiveram-se 186 linhas temáticas de pesquisa sobre covid-19 e pós-covid, as quais foram categorizadas conforme os eixos da COVID-END. Após a análise, o agrupamento por semelhança e a exclusão de respostas, gerou-se a lista de 161 linhas temáticas, sendo 42 no Eixo I – Medidas de saúde pública; 45 no Eixo II – Manejo clínico de covid-19 e questões de saúde relacionadas à pandemia; 32 no Eixo III – Organização dos sistemas de saúde para a covid-19; e 42 no Eixo IV – Respostas econômicas e sociais. 

Das 161 linhas temáticas de pesquisa avaliadas pelo painel de especialistas na rodada 1 do método Delphi, 139 obtiveram o percentual de aprovação positiva igual ou maior que 70,0%. Nessa rodada, os especialistas propuseram a inclusão de mais 51 linhas temáticas de pesquisa. Após a análise e sistematização, essas novas propostas resultaram em 41 novas linhas temáticas, as quais foram inseridas nos questionários eletrônicos para serem avaliadas na rodada 2.

Na segunda rodada, 63 linhas temáticas de pesquisa foram avaliadas, decorrentes das 22 que não alcançaram o consenso, e 41 novas linhas foram adicionadas pelo painel de especialistas na primeira rodada. Das 63 linhas temáticas examinadas, 40 obtiveram o consenso (aprovação positiva igual ou maior que 70,0%). Foi gerado o conjunto de 179 linhas temáticas de prioridades de pesquisa em covid-19 e seus efeitos. Apresentou-se o resultado do consenso das primeira e segunda rodadas entre as linhas temáticas de pesquisa em cada eixo da COVID-END (Tabelas 4, 5, 6 e 7).

Foram elencadas 50 linhas prioritárias nos três temas do Eixo I – Medidas de saúde pública (Tabela 2). A maioria das linhas se concentrou no tema: “Medidas amplas de saúde pública” com 31 linhas (62,0%). Nos três temas, foram frequentes as linhas sobre vacinas preventivas para a covid-19. Das 50 propostas avaliadas, 76,0% alcançaram o consenso na primeira rodada.

**Tabela 2:** Resultado do consenso das primeira e segunda rodadas do método, classificado nos temas do Eixo I – Medidas de saúde pública. Brasil, 2022-2023

Temas de pesquisa	Linhas de pesquisa	Primeira rodada^a^	Segunda rodada	nº de linhas temáticas
		Consenso(%)	Consenso(%)	
**Eixo I**	**Medidas de saúde pública**	(n=5)	(n=5)	
**Prevenção da infecção**	Manutenção da vacinação anual para a covid-19.	100	-	12
	Estratégias para o aumento da cobertura vacinal.	100	-	
	Avaliar a distribuição de vacinação completa por indicadores demográficos, geográficos e socioeconômicos.	100	-	
	Impacto da vacinação na incidência da forma grave da covid-19.	100	-	
	Análise das causas da redução da vacinação para covid-19.	-	100	
	Qualidade e oferta de acesso às vacinas para crianças e jovens.	-	80	
	Análise do acesso à vacina e os determinantes sociais com recorte de gênero e raça.	-	100	
	Avaliação e acompanhamento da cobertura vacinal em comunidades tradicionais e quilombolas.	-	100	
	Efetividade e impacto da adesão de medidas preventivas contra a covid-19 para diferentes públicos.	100	-	
	Análise das estratégias de prevenção e comunicação para a covid-19 desenvolvidas por movimentos sociais e instituições de pesquisa.	100	-	
	Intervenções em educação no controle da covid-19.	100	-	
	Avaliação da eficiência das estratégias de educação e comunicação em saúde em tempos de pandemia.	100	-	
**Controle da infecção**	Avaliação da divulgação dos efeitos positivos das vacinas para a covid-19.	100	-	7
	Avaliação do impacto da covid-19 utilizando o Sistema de Informação de Mortalidade.	100	-	
	Avaliação do acesso de medicamentos de alto custo para a covid-19.	60	80	
	Avaliação do acesso e da implementação de programas de testagem e monitoramento do SARS-CoV-2 e variantes genéticas na rede de atenção à saúde, áreas urbanas e rurais.	100	-	
	Desenvolvimento de métodos práticos e eficazes de desinfecção do ar em ambientes fechados e superfícies.	80	-	
	Medidas comportamentais mais eficazes (epidemiológicas e socioculturais) de controle da infecção por covid-19.	100	-	
	Avaliação de mudanças de comportamento e estilos de vida após a pandemia.	100	-	
**Medidas amplas de saúde pública**	Avaliação do acesso a vacinação para covid-19 na Atenção Primária a Saúde.	100	-	31
	Efeitos colaterais da vacina de covid-19.	-	80	
	Avaliação da Política Nacional de Imunização brasileira e sua relação com a cobertura vacinal da covid-19.	100	-	
	Relevância das pesquisas e atividades realizadas por instituições públicas decorrentes da covid-19.	80	-	
	Financiamento do fomento em pesquisas para a prevenção e vigilância para covid-19.	80	-	
	Avaliação do financiamento dos órgãos de fomento, indústrias e instituições doadoras para pesquisa, desenvolvimento e inovação de insumos essenciais para a covid-19.	80	-	
	Avaliação de metodologias e intervenções para qualificar profissionais para a prevenção e controle da covid-19 e pós-covid.	80	-	
	Impactos da covid-19 no adoecimento e no processo de trabalho dos profissionais da saúde.	100	-	
	Avaliação de campanhas de informação e prevenção sobre covid-19 em mídias sociais e outros canais de comunicação.	100	-	
	Avaliação da participação social em campanhas de informação sobre covid-19.	100	-	
	Consequências da infodemia e comunicação em saúde para covid-19.	80	-	
	Avaliação de impacto da covid-19 na saúde da população.	100	-	
	Identificação de grupos mais vulneráveis ao agravamento e óbito por covid-19.	100	-	
	Análise da morbimortalidade da covid-19 na população negra.	-	100	
	Impactos da pandemia da covid-19 na saúde da criança e do adolescente.	80	-	
	Avaliação de impacto da covid-19 na saúde psicossocial e mental da população.	100	-	
	Avaliação da utilização dos serviços de saúde para a covid-19.	100	-	
	Analisar os efeitos da covid-19 nas comunidades amazônicas.	100	-	
	Monitoramento e levantamento de reservatórios naturais do vírus da família SARS-CoV-2 nos neotrópicos e em ambientes urbanos.	100	-	
	Efeitos colaterais da vacina para covid-19 na redução de acuidade visual.	80	-	
	Efeitos da covid-19 nas doenças preexistentes em pessoas com deficiência.	60	100	
	Efeitos pós-covid em pessoas com deficiências.	60	80	
	Implementação da rede de atenção especializada para pessoas com sequelas pós-covid.	100	-	
	Pesquisas ecológicas sobre internações sensíveis à Atenção Primária à saúde e covid-19 e pós-covid.	60	100	
	Avaliação do acompanhamento pelas equipes de Saúde da Família e Atenção Especializada para reabilitação dos casos de pós-covid.	100	-	
	Avaliação da incidência e características sociodemográficas de pessoas com pós-covid.	100	-	
	Pós-covid em crianças e adolescentes.	-	100	
	Fatores de risco biológicos e sociais associados a pós-covid.	100	-	
	Estudo de avaliação para a carga da síndrome pós-covid no Brasil.	100	-	
	Uso de recursos rápidos e disponíveis para o desenvolvimento de aparelhos e equipamentos no tratamento de covid-19.	80	-	
	Análise dos dados desagregados por raça e cor no sistema de informação de vacinação.	-	100	

^a^Na coluna: primeira rodada, o símbolo (-) indica que as linhas temáticas de pesquisa foram avaliadas na segunda rodada.

Observaram-se as 45 linhas temáticas de pesquisa avaliadas no Eixo II – Manejo clínico de covid-19 e questões de saúde relacionadas à pandemia, das quais 91,0% atingiram o consenso na primeira rodada (Tabela 3). Destacaram-se os temas “Profilaxia para covid-19” e “Manejo clínico dos impactos relacionados à pandemia”, com o maior número (10 linhas, 22,0%). As propostas desses temas estavam mais concentradas em vacinas e suporte clínico para pacientes internados com covid-19. Em “Gestão da covid-19 com enfoque sindêmico” (4 linhas), a maioria das propostas relacionou-se com os impactos da doença nas periferias brasileiras. Nos temas “Tratamento para condições pós-covid” e “Manejo clínico dos impactos relacionados à pandemia” (8 e 10 linhas), os participantes fizeram propostas sobre reabilitação física, psicológica, neurológica e pós-covid em pacientes imunossuprimidos, bebês e crianças. Em “Promoção da saúde para covid-19” (4 linhas), citaram-se linhas temáticas sobre impacto do exercício físico, saúde mental dos profissionais da saúde e efeitos negativos da infodemia.

**Tabela 3:** Resultado do consenso da primeira e segunda rodadas do método Delphi, classificado nos temas do Eixo II – Manejo clínico de covid-19 e questões de saúde relacionadas à pandemia. Brasil, 2022-2023

Temas de pesquisa	Linhas de pesquisa	Primeira rodada^a^	Segunda rodada	nº de linhas temáticas
		Consenso (%)	Consenso (%)	
**Eixo II**	**Manejo clínico de covid-19 e questões de saúde relacionadas à pandemia**	(n=6)	(n=6)	
**Profilaxia para covid**-19	Atualização e desenvolvimento de vacinas preventivas e terapêuticas com novas tecnologias para as variantes virais.	100	-	10
	Resposta imunológica da vacina nos grupos de risco.	100	-	
	Avaliação de resposta vacinal em crianças e jovens.	-	100	
	Desenvolvimento de ensaios clínicos multicêntricos de fase III para novas vacinas.	100	-	
	Estratégias para a ampliação do número de doses da vacina de imunização para a covid-19.	83	-	
	Disponibilidade de imunobiológicos para crianças e jovens com doenças crônicas.	-	83	
	Desenvolvimento de insumos para a fabricação de vacinas no Brasil.	100	-	
	Desenvolvimento de insumos de diagnóstico para covid-19 e outras doenças endêmicas e epidêmicas no Brasil.	100	-	
	Desenvolvimento de novos medicamentos para covid-19 e pós exposição ao SARS-CoV-2.	83	-	
	Desenvolvimento de medicamentos de ampliação da proteção à covid-19 para pacientes imunossuprimidos.	83	-	
**Tratamento clínico da covid**-19	Estudos de coorte histórica para avaliação da efetividade dos tratamentos para a covid-19 a base de corticoide e antivirais.	83	-	9
	Avaliação dos eventos adversos às vacinas.	-	100	
	Uso de medicamentos antivirais - Paxlovid (nirmatrelvir + ritonavir) e molnupiravir em pacientes com covid-19 em contexto ambulatorial.	83	-	
	Efetividade de tratamento de covid-19 para imunossuprimidos e grupos de risco.	100	-	
	Implementação e avaliação de protocolos clínicos e diretrizes terapêuticas para tratamento domiciliar, hospitalar (UTI e síndrome pós-terapia-intensiva) e pós-covid-19.	100	-	
	Revisões sistemáticas sobre a efetividade das estratégias de tratamentos para covid-19.	100	-	
	Mecanismos seguros de atendimento em pacientes assintomáticos: riscos e protocolos assistenciais.	83	-	
	Pesquisas sobre os cuidados de suporte para pacientes com sintomas neurológicos da covid-19.	83	-	
	Estudo qualitativo sobre o atendimento nos serviços de saúde pós-alta hospitalar de pessoas internadas por covid-19 em unidades de terapia intensiva.	83	-	
**Gestão da covid-19 com enfoque sindêmico**	Estudos da vigilância dos óbitos por SARS-CoV-2 com ênfase na definição dos critérios para classificação e integração entre os sistemas de informações de notificação e mortalidade.	100	-	4
	Assistência à saúde mental nas periferias brasileiras no contexto da covid-19.	100	-	
	Organização e qualidade do cuidado nas periferias brasileiras no contexto da covid-19 e pós-covid.	100	-	
	Estudos sobre interrelações entre covid-19 e outras doenças infecciosas endêmicas no longo prazo.	100	-	
**Tratamento para condições pós**-covid	Efetividade de práticas para a reabilitação física e mental de pacientes pós-covid.	100	-	8
	Efeitos e necessidades psicológicas nos pacientes com covid-19.	100	-	
	Compreensão de manifestações neurológicas e manejo clínico pós-covid.	100	-	
	Seguimento da evolução de sequelas específicas da covid-19 (dispneia, déficit de memórias, insônia, depressão).	100	-	
	Avaliação prospectiva de pessoas recuperadas de covid-19 com abordagem clínico-laboratorial.	83	-	
	Efetividade de tratamento no pós-covid para imunossuprimidos e grupos de risco.	100	-	
	Sequelas e efeitos do pós-covid em bebês e crianças.	100	-	
	Diagnóstico da rede especializada nos municípios para atenção de patologias por internação em unidades intensivas e pós-covid.	83	-	
**Manejo clínico dos impactos relacionados à pandemia**	Inquérito sobre manejos clínicos adotados nos estados brasileiros para a covid-19.	83	-	10
	Avaliação dos fluxos de atendimentos durante a pandemia de covid-19.	100	-	
	Avaliação do acesso ao seguimento clínico de sequelas da covid-19.	83	-	
	Melhores práticas de intervenção psicológica para atender as sequelas psicossociais da covid-19.	100	-	
	Avaliar manejos clínicos eficientes para pacientes com sequelas pulmonares da covid-19.	100	-	
	Estudos sobre manejo clínico de deficiências imunológicas e câncer e a interrelação com as infeções e reinfecções por covid-19.	83	-	
	Impacto da pandemia de covid-19 na incidência e mortalidade por diferentes causas.	100	-	
	Impacto da pandemia de covid-19 no diagnóstico, acompanhamento e tratamento por diferentes causas.	100	-	
	Impacto da pandemia de covid-19 sobre a natalidade e saúde sexual e reprodutiva.	83	-	
	Condições de saúde negligenciadas com a pandemia de covid-19.	83	-	
**Promoção da saúde para covid**-19	Identificação das melhores intervenções de cuidado para a manutenção da saúde.	67	100	4
	Impacto do exercício físico como medida de promoção de saúde e prevenção da covid-19.	100	-	
	Estudos observacionais associados a saúde mental de profissionais de saúde com e sem carga de trabalho com pacientes com covid-19.	83	-	
	Efeitos negativos da infodemia na promoção da saúde para a covid-19.	50	83	

^a^Na coluna primeira rodada, o símbolo (-) indica que as linhas temáticas de pesquisa foram avaliadas na segunda rodada.

O Eixo III – Organização dos sistemas de saúde para a covid-19, com 35 linhas, teve o maior número de propostas nos temas “Organização da prestação de serviços para a covid-19” (11 linhas, 31,0%) e “Arranjos de governança para a covid-19” (10 linhas, 29,0%) (Tabela 4). Nos três primeiros temas “Arranjos transversais que precisam de enfoque sistêmico para a covid-19”, “Organização da prestação de serviços para a covid-19” e “Arranjos financeiros para a covid-19”, a atenção dos participantes do estudo estava voltada para temas relacionados com saúde mental, crianças e imunossuprimidos, organização e repasse de recursos financeiros nos três níveis de atenção à saúde. Em “Arranjos de governança para a covid-19” (quem pode tomar quais decisões), prevaleceram temas relacionados com a resposta do governo federal mediante a tomada de decisão para a pandemia de covid-19. Nesse eixo, 71,0% das linhas temáticas atingiram o consenso logo na primeira rodada. 

**Tabela 4:** Resultado do consenso da primeira e segunda rodadas do método Delphi, classificado nos temas do Eixo III – Organização dos sistemas de saúde para a covid-19. Brasil, 2022-2023

Temas de pesquisa	Linhas de pesquisa	Primeira rodada^a^	Segunda rodada	nº de linhas temáticas
		Consenso (%)	Consenso (%)	
**Eixo III**	**Organização dos sistemas de saúde para a covid**-19	(n=5)	(n=5)	
**Arranjos transversais que precisam de enfoque sistêmico para a covid**-19	Enfoque sistêmico para a covid-19, comorbidades e hábitos de alimentação, nutrição e atividade física.	80	-	7
	Enfoque sistêmico para a covid-19 e saúde mental.	100	-	
	Enfoque sistêmico para covid-19 e crianças.	-	80	
	Enfoque sistêmico para covid-19 e imunossuprimidos.	-	80	
	Avaliação do sistema de notificação obrigatória de casos de síndrome gripal.	-	80	
	Modelos de intervenções online em saúde mental para pacientes pós-covid.	100	-	
	Análise da preparação, resposta e capacidade de resiliência do sistema de saúde brasileiro a emergências de saúde pública.	100	-	
**Organização da prestação de serviços para a covid**-19	Organização da rede de atenção à saúde (primária, média e alta complexidade) para a pós-covid.	100	-	11
	Impactos da covid-19 na utilização de serviços ambulatoriais, hospitalares e de atenção primária à saúde para diferentes causas.	100	-	
	Avaliação da adequação da infraestrutura na rede de saúde para covid-19.	80	-	
	Planejamento da demanda de serviços de saúde primários, secundários e terciários para enfrentamento da covid-19 nas três esferas de governo.	100	-	
	Avaliação dos serviços governamentais de provisão dos leitos e insumos essenciais nos três níveis de atenção à saúde, no enfrentamento da covid-19.	100	-	
	Avaliação dos hospitais de campanha criados na pandemia de covid-19.	-	80	
	Impactos da covid-19 na estrutura e organização da Atenção Primária à Saúde.	60	100	
	Organização da rede de serviços da saúde suplementar e privada para a covid-19.	80	-	
	Avaliação do uso dos equipamentos de saúde para pacientes pós-covid e populações vulneráveis.	60	80	
	Impactos da covid-19 nos pacientes não covid-19 nas filas de espera e morbimortalidade para cirurgias eletivas das diversas especialidades.	100	-	
	Avaliação dos resultados da mortalidade e letalidade por covid-19 nos diferentes modelos assistenciais implantados nos municípios do sistema público.	80	-	
**Arranjos financeiros para a covid**-19	Avaliação do custo-benefício para aquisição e uso de testes rápidos de antígenos para casos leves de covid-19.	-	80	7
	Avaliação de impacto das transferências de recursos financeiros federais para estados e municípios na resposta a pandemia de covid-19.	100	-	
	Impactos financeiros da covid-19 na saúde pública.	100	-	
	Recursos financeiros e de profissionais treinados para reabilitação e reinserção social de pessoas com pós-covid.	80	-	
	Incentivos financeiros para ampliação do acesso aos serviços especializados para pacientes com síndrome pós cuidados intensivos da covid-19 nos municípios.	100	-	
	Acesso de populações vulneráveis aos medicamentos contra a covid-19 aprovados pela Organização Mundial da Saúde.	60	100	
	Custo-efetividade do tratamento da covid-19 e pós-covid.	80	-	
**Arranjos de governança para a covid-19** (**quem pode tomar quais decisões**)	Estudos de caso da resposta de secretarias estaduais e municipais de saúde diante da covid-19.	100	-	10
	A intervenção do Estado na vida privada em tempos de covid-19.	80	-	
	Utilização das recomendações da Organização Mundial da Saúde pelo governo federal.	80	-	
	Estudos comparativos entre governo federal e municipais para medidas de controle da covid-19 em áreas urbanas.	60	80	
	Pacto federativo e Programa Nacional de Imunizações.	100	-	
	Estudo de benchmarking de práticas de governança para a covid-19 em outros países.	80	-	
	Participação das câmaras técnicas e comitês como espaços de construção de consensos e decisões na crise sanitária da covid-19.	80	-	
	Avaliação da participação de especialistas e dos conselhos de saúde nas tomadas de decisão durante e pós surtos da covid-19.	80	-	
	Crise Sanitária e Seguridade Social.	80	-	
	Impactos e influência do governo federal na organização do SUS e no uso de precauções para prevenção e enfrentamento da covid-19.	60	80	

^a^Na coluna primeira rodada, o símbolo (-) indica que as linhas temáticas de pesquisa foram avaliadas na segunda rodada.

No Eixo IV – Respostas econômicas e sociais, elencaram-se 49 prioridades de pesquisa. Os temas com maior destaque foram educação (8 linhas, 16,0%) e trabalho (7 linhas, 14,0%). Nesses temas, o maior número de linhas temáticas apontado pelos respondentes se refere a medicamentos, serviços de saúde, governança do sistema de saúde, educação e trabalho relacionados à covid-19 (Tabela 5).

**Tabela 5:** Resultado do consenso da primeira e segunda rodadas do método Delphi, classificado nos temas do Eixo IV – Respostas econômicas e sociais. Brasil, 2022-2023

Temas de pesquisa	Linhas de pesquisa	Primeira rodada^a^	Segunda rodada	nº de linhas temáticas
		Consenso (%)	Consenso (%)	
**Eixo IV**	**Respostas econômicas e sociais**	(n=4)	(n=3)	
**Serviços para crianças** **e jovens**	Consequências do isolamento social para recém-nascidos e crianças menores de cinco anos na pandemia de covid-19.	100	-	2
	Sociabilidade de crianças e jovens e saúde mental no contexto da pós-pandemia de covid-19.	100	-	
Cidadania	Impacto da cultura e da cidadania no enfrentamento da covid-19.	100	-	2
	Prestação e evolução dos serviços públicos na pandemia de covid-19.	100	-	
**Serviços sociais e** comunitários	Efeitos da covid-19 na prestação de serviços sociais e comunitários no acolhimento de pessoas em situação de vulnerabilidade.	100	-	5
	Participação das organizações da sociedade civil no combate a covid-19.	100	-	
	Avaliação de projetos de apoio biopsicossocial realizados pelo terceiro setor e métodos inovadores para sua disseminação.	100	-	
	Acesso dos moradores das periferias às informações sobre covid-19.	-	100	
	Disponibilidade e oferta de kits de higiene (máscara, álcool em gel) para as comunidades periféricas.	-	100	
Cultura **e gênero**	Impacto da covid-19 na divisão sexual do trabalho e saúde.	75	-	3
	Desenvolvimento e disseminação de atividades culturais inovadoras e à distância adequadas para diferentes faixas etárias pós-pandemia de covid-19.	75	-	
	Impacto da covid-19 nas desigualdades de gênero.	75	-	
**Desenvolvimento e crescimento econômico**	Contribuições das organizações da sociedade civil para o desenvolvimento e crescimento econômico de comunidades vulneráveis para proteção da covid-19.	100	-	3
	O papel do Estado nas empresas privadas e no comércio na pandemia de covid-19.	100	-	
	Respostas econômicas e sociais das consequências do desestímulo e do atraso na ampla vacinação da população brasileira contra a covid-19.	100	-	
Educação	Avaliação do impacto da pandemia de covid-19 na aprendizagem nas escolas públicas de ensino fundamental e médio nos municípios.	100	-	8
	Desenvolvimento de métodos inovadores de ensino à distância para o ensino fundamental, médio e superior.	100	-	
	Impacto das tecnologias de ensino remoto na docência.	-	100	
	Evasão e repetência escolar em tempos de covid-19.	-	100	
	Avaliação do desemprego docente na pandemia e pós- pandemia.	-	100	
	Contribuição das organizações da sociedade civil no desenvolvimento de plataformas para programas e projetos de educação.	100	-	
	Impactos da covid-19 no trabalho docente no ensino fundamental, médio e superior e saúde mental.	100	-	
	Intervenções para enfrentar as perdas causadas pela crise sanitária da covid-19 na educação pública.	100	-	
Trabalho	Perda de função vital e de trabalho relacionada à covid-19.	100	-	7
	Ideação suicida e suicídio em trabalhadores/as do agronegócio na pandemia de covid-19.	100	-	
	Análise da oferta de emprego antes e após a pandemia covid-19.	-	100	
	Impacto da covid-19 no segmento das empregadas domésticas e outros profissionais de serviços gerais.	-	100	
	Impacto da crise sanitária pela covid-19 nas políticas de emprego e renda do Brasil.	100	-	
	Analisar o efeito da covid-19 no mercado de trabalho formal e informal em diferentes setores da economia.	100	-	
	Estudos sobre vulnerabilidade social e impacto de ausência de medidas de mitigação no mundo do trabalho e da educação.	100	-	
**Proteção financeira**	Avaliação de propostas de transferência de renda e renda mínima para pessoas afetadas na sua atividade laboral pela pandemia.	100	-	6
	Avaliação da cobertura e focalização de programas governamentais de proteção financeira para compensar as perdas de renda pela covid-19.	75	-	
	Necessidades de suporte econômico e social para crianças e jovens com perdas familiares por covid-19 e pessoas com pós-covid.	100	-	
	A covid-19 e o impacto de políticas de renda mínima nos determinantes sociais de saúde.	75	-	
	Avaliação do impacto das sequelas da covid-19 na renda das famílias em situação de vulnerabilidade social.	100	-	
	Impacto da mortalidade por covid-19 na população economicamente ativa.	100	-	
**Segurança alimentar e alimentação segura**	Segurança alimentar municipal, estadual e nacional antes e durante a pandemia de covid-19.	100	-	3
	Avaliação da cobertura e focalização de programas de distribuição de cestas básicas e refeições prontas decorrentes das perdas de renda familiar.	100	-	
	Garantia de alimentação segura para pessoas com renda menor de um salário-mínimo.	100	-	
Habitação	Desenvolvimento de projetos arquitetônicos inovadores para garantir ventilação adequada nas habitações individuais (casas) e coletivas (prédios).	50	100	4
	Alternativas de moradia para a população em situação de rua.	-	100	
	Habitação e covid-19: efeitos das condições precárias de moradia e a disseminação da covid-19.	-	100	
	Impactos de projetos governamentais e sociais para a promoção da habitação social.	-	100	
Infraestrutura	Análise das condições urbanas e expansão do vírus SARS-CoV-2.	100	-	1
Transporte	Lotação do transporte público e os riscos de contaminação e infecção por covid-19.	100	-	
	Desenvolvimento de métodos práticos e eficazes de desinfecção dos veículos de uso coletivo.	100	-	
	Impacto no planejamento ou modelo de transporte público pós-pandemia de covid-19.	100	-	5
	Transporte público e covid-19: ações dos governos municipais, estaduais e federal e das empresas privadas para conter a disseminação.	-	100	
	Impacto no setor transporte durante e pós pandemia de covid-19.	-	100	

^a^Na coluna primeira rodada, o símbolo (-) indica que as linhas temáticas de pesquisa foram avaliadas na segunda rodada.

A similaridade de termos dos temas verificada através de análise de nuvem de palavras evidenciou que as linhas de pesquisas relacionadas com impacto e avaliação foram preocupação transversal nos quatro eixos, entre os participantes deste estudo. Assim como o pós-covid que aparece nos quatro eixos da COVID-END, indicou-se que constitui prioridade de pesquisa entre os participantes deste estudo.

No conjunto da análise, destacaram-se as doenças mentais e em situação de imunossupressão, identificadas no Eixo II – Manejo clínico de covid-19 e questões de saúde relacionadas à pandemia. As linhas de pesquisa sobre doenças mentais foram relacionadas com os efeitos nas periferias brasileiras, na reabilitação de pacientes pós-covid e nos profissionais de saúde. As doenças em situação de imunossupressão estão relacionadas com pesquisas sobre efetividade do tratamento durante e no pós-covid.

Considerando a repetição de palavras em três dos quatro eixos, a população prioritária entre os participantes do estudo foram crianças. As linhas prioritárias referentes a esse grupo foram relacionadas com pós-covid, qualidade e oferta de vacinas, resposta vacinal, imunobiológicos para doenças crônicas, consequências do isolamento social, sociabilidade no contexto pós-pandêmico e suporte econômico para crianças com perdas familiares decorrentes da covid-19.

## Discussão

Este estudo identificou as prioridades de pesquisa em covid-19 e pós-covid-19 na perspectiva de atores sociais das áreas de conhecimentos que contemplam das condições clínicas, da magnitude e determinantes sociais e dos impactos sociais e econômicos da covid-19. Entre as linhas de pesquisa elencadas por consenso entre os(as) participantes, predominaram estudos de avaliação, impactos sociais e de saúde da covid-19, pós-covid e suas sequelas. As doenças mentais e em situação de imunossupressão destacaram-se no conjunto de doenças. A população com maior interesse entre os participantes foi a de crianças. Alguns dos temas prioritários de pesquisa apontaram estudos sobre medicamentos, respostas dos serviços de saúde, governança do sistema de saúde, efeitos da covid-19 na educação e trabalho.

As limitações do estudo referem-se à baixa adesão e à pouca diversidade do perfil das(os) participantes, especialmente gestoras(es) e usuários do SUS. Os temas apresentados podem não abranger amplamente os impactos da covid-19 e suas sequelas na população brasileira, visto que a pandemia afetou de diferentes formas os grupos sociais. Este estudo foi concluído no período de um ano, em que a magnitude da pandemia de covid-19 mudou no país e novas intervenções foram necessárias para enfrentar as suas consequências. A utilização dos temas prioritários de pesquisa deve considerar essas mudanças e a necessidade de realizar o ranqueamento de priorização no curto, médio e longo prazo.

Foram vários os impactos que a covid-19 causou nos diversos âmbitos da sociedade. Nos serviços de saúde, o constante aumento dos casos da doença, entre 2020 e 2021, provocou colapsos nos sistemas de saúde e pesquisa no mundo, forçando a reorganização em larga escala de hospitais e a melhoria da infraestrutura e dos recursos financeiros no SUS (26).

A pandemia afetou a educação com mudanças no processo de aprendizagem nas escolas. O Brasil foi um dos países que passou mais tempo com escolas fechadas devido ao isolamento social e teve como alternativa o ensino remoto (27). No mercado de trabalho brasileiro, observou-se que, nos primeiros meses de pandemia, as taxas de desemprego aumentaram, e ocorreu a migração dos trabalhadores de empregos formais para informais (28). Os impactos da covid-19 mostraram-se relevantes em nível nacional e internacional para as agendas de prioridades de pesquisa sobre covid-19. O estudo de prioridades de pesquisa da covid-19 no Sudeste Asiático também foi apresentado como temática (5,11-13).

As manifestações clínicas do vírus e os sintomas recorrentes ou persistentes após a infecção foram denominados “condições pós-covid-19” ou “covid longa” (29). Em 2021, dos 1.790.796 óbitos registrados no Brasil, 2.948 eram decorrentes de causas pós-covid, cerca de 1,6 óbito por causa de pós-covid-19 para cada 1 mil registros. Entre março e abril de 2022, em 720 indivíduos que testaram positivo para covid-19, 69,0% apresentaram sintomas por mais de três meses a partir do início do quadro agudo da doença (30-31).

A saúde mental foi destacada como prioridade de pesquisa devido aos impactos psicológicos e sociais da pandemia, evidenciados desde suas primeiras semanas (32). Há preocupação com o impacto da covid-19 em populações de risco, especialmente em indivíduos imunocomprometidos, que têm maior vulnerabilidade a infecções e complicações graves (33). Torna-se fundamental voltar a atenção do desenvolvimento em pesquisas nessas áreas. 

Embora o risco de casos graves e mortalidade por covid-19 seja menor em crianças, elas podem evoluir para quadros críticos. A falta de inclusão dessa população no desenvolvimento inicial de tratamentos é preocupante (34-35). Além da patologia, as implicações do isolamento social para crianças ocasionaram o rompimento brusco da maioria de interações humanas presenciais, causando o uso excessivo da internet, muitas vezes sem monitoramento dos pais ou tutores, o que colocou crianças e adolescentes nas mais diversas situações de vulnerabilidade (36). Embora existam estudos sobre o impacto da pandemia em crianças, ainda existem lacunas de conhecimento consideráveis nesse tema (12-13). É fundamental garantir a priorização de pesquisas em saúde, educação e sociabilidade para o desenvolvimento de ações e estratégias para essa população.

A covid-19 e pós-covid impactam saúde, vida e convívio social, destacando a necessidade de pesquisas para soluções. A agenda de prioridades orienta investimentos, monitora resultados e atualiza ações conforme a situação de saúde.

A utilização dos quatro eixos da iniciativa COVID-END na formulação dos questionários foi um ponto forte do estudo, facilitando a condução online e abrangente com diversidade de participantes. A iniciativa foi voltada para a tomada de decisões sobre a covid-19 e abordava temas como prevenção, manejo clínico, organização dos sistemas de saúde e respostas econômicas e sociais, sendo um dos 50 principais grupos globais de síntese de evidências (20). 

Outro ponto a ser salientado foi a utilização da metodologia que se consolidou em duas etapas: i) a consulta ampla que possibilitou o levantamento de linhas prioritárias de pesquisa, a partir da composição e do processo de seleção de pesquisadores(as) de diferentes áreas de conhecimento, gestores(as) e profissionais de saúde com experiência direta e conhecimento na gestão e assistência durante e após a pandemia, gestores(as) de ciência e tecnologia que lançaram editais sobre a covid-19 e usuários(as) do SUS; e ii) a definição de consenso da priorização pelo método Delphi com um painel de especialistas em covid-19.

A definição de prioridades de pesquisa sobre covid-19 e pós-covid-19, sem restrição prévia de tipo de investigação, facilita o financiamento de diversas abordagens. Isso garante que políticas e programas sejam baseados em evidências, preenchendo lacunas de conhecimento, prevenindo crises futuras e promovendo pesquisas multidisciplinares e complementares (5,37).

Os resultados são importantes para países como o Brasil, que têm recursos limitados para pesquisa, sendo essencial alocar esses recursos de forma eficiente. A priorização de pesquisas durante a pandemia foi adotada por diversos países, como Irã e nações da Ásia e América Latina, alinhando as prioridades às necessidades locais (5,38-39). 

A produção científica eficaz em epidemias pode ser alcançada com estruturas de pesquisa multidisciplinares, regulação, financiamento, parcerias e confiança sustentáveis, mantidas nos períodos entre epidemias (10). Ressalta-se que esta não é uma agenda acabada e nem deve ser, pois precisa de esforços contínuos e atualização permanente de acordo com o contexto sanitário e epidemiológico da covid-19 e as consequências pós-covid.

O financiamento de pesquisas alinhadas à priorização é estratégia eficiente para fortalecer a ciência e tecnologia, além de ajudar na formulação de políticas públicas, especialmente para populações vulneráveis, que são as mais afetadas em emergências.

## Data Availability

O banco de dados e os códigos de análise utilizados na pesquisa estão disponíveis em: https://docs.google.com/spreadsheets/d/1C2hU3oqWFJe8VSiLpdhVfpVT82yhlQrQ/edit?gid=1623398596#gid=1623398596
